# Tissue Distribution and Metabolization of Ciguatoxins in an Herbivorous Fish following Experimental Dietary Exposure to *Gambierdiscus polynesiensis*

**DOI:** 10.3390/md22010014

**Published:** 2023-12-25

**Authors:** Rachel J. Clausing, Hela Ben Gharbia, Khalil Sdiri, Manoëlla Sibat, Ma. Llorina Rañada-Mestizo, Laura Lavenu, Philipp Hess, Mireille Chinain, Marie-Yasmine Dechraoui Bottein

**Affiliations:** 1 Dipartimento di Scienze della Terra dell’Ambiente e della Vita, Università degli Studi di Genova, 16132 Genova, Italy; 2 Department of Ecology and Evolutionary Biology, University of California, Los Angeles, CA 90095, USA; 3 IAEA Marine Environment Laboratories, International Atomic Energy Agency, 98000 Monaco, Monaco; helabengharbia.hbg@gmail.com (H.B.G.); k.sdiri@iaea.org (K.S.); laura.lavenu@hotmail.com (L.L.); 4 Ifremer, ODE/PHYTOX/METALG, Rue de l’île d’Yeu, F-44300 Nantes, France; manoella.sibat@ifremer.fr (M.S.); philipp.hess@ifremer.fr (P.H.); 5 IAEA Collaborating Center on Harmful Algal Bloom (HAB) Studies, Chemistry Research Section, Department of Science and Technology, Philippine Nuclear Research Institute (DOST-PNRI), Diliman, Quezon City 1101, Philippines; mlrmestizo@pnri.dost.gov.ph; 6 Laboratoire des Biotoxines Marines, UMR 241 EIO, Institut Louis Malardé, BP 30, Papeete-Tahiti 98713, French Polynesia; mchinain@ilm.pf; 7 Université Côte d’Azur, CNRS, ECOSEAS, UMR7035, Parc Valrose, CEDEX 2, 06103 Nice, France; marie-yasmine.bottein@univ-cotedazur.fr

**Keywords:** ciguatoxins, ciguatera poisoning (CP), trophic transfer, metabolism, biotransformation, bioaccumulation, tissue distribution, *Gambierdiscus polynesiensis*, reef fish, liquid chromatography–tandem mass spectrometry

## Abstract

Ciguatoxins (CTXs), potent neurotoxins produced by dinoflagellates of the genera *Gambierdiscus* and *Fukuyoa*, accumulate in commonly consumed fish species, causing human ciguatera poisoning. Field collections of Pacific reef fish reveal that consumed CTXs undergo oxidative biotransformations, resulting in numerous, often toxified analogs. Following our study showing rapid CTX accumulation in flesh of an herbivorous fish, we used the same laboratory model to examine the tissue distribution and metabolization of Pacific CTXs following long-term dietary exposure. *Naso brevirostris* consumed cells of *Gambierdiscus polynesiensis* in a gel food matrix over 16 weeks at a constant dose rate of 0.36 ng CTX3C equiv g^−1^ fish d^−1^. CTX toxicity determination of fish tissues showed CTX activity in all tissues of exposed fish (eight tissues plus the carcass), with the highest concentrations in the spleen. Muscle tissue retained the largest proportion of CTXs, with 44% of the total tissue burden. Moreover, relative to our previous study, we found that larger fish with slower growth rates assimilated a higher proportion of ingested toxin in their flesh (13% vs. 2%). Analysis of muscle extracts revealed the presence of CTX3C and CTX3B as well as a biotransformed product showing the *m*/*z* transitions of 2,3-dihydroxyCTX3C. This is the first experimental evidence of oxidative transformation of an algal CTX in a model consumer and known vector of CTX into the fish food web. These findings that the flesh intended for human consumption carries the majority of the toxin load, and that growth rates can influence the relationship between exposure and accumulation, have significant implications in risk assessment and the development of regulatory measures aimed at ensuring seafood safety.

## 1. Introduction

Ciguatoxins (CTXs) are potent neurotoxins produced by epiphytic dinoflagellates of the genera *Gambierdiscus* and *Fukuyoa* [1,2,3] that cause human ciguatera poisoning (CP), the most frequently reported non-bacterial seafood-borne illness globally [4,5,6]. These dinoflagellates that produce CTXs inhabit tropical and subtropical coastal reefs worldwide [7,8], but have increasingly been found in cooler more temperate waters [9,10] (e.g., Canary Islands [11], temperate Japan [12], New Zealand [13], Crete [9], and the Balearic Islands [14]). Correspondingly, cases of ciguatera also appear to be increasing in occurrence [5,15,16], due not only to climate change-associated range expansion [8], but also to expanding tourism and fish trade [10,17,18].

CTXs enter tropical and sub-tropical food webs through grazing by herbivores or detritivores of host macroalgae or benthos inhabited by the toxin-producing cells. CTXs are subsequently transferred along the food chain [19,20] and may cause CP when toxin-containing marine organisms are consumed [21]. Although higher-order commonly consumed fish species are usually associated with CP (e.g., groupers, snappers, jacks, mackerels) [22,23], CTXs are now known to occur throughout coral reef food webs, including in herbivorous and omnivorous fish and invertebrates [24,25,26,27,28]. *Gambierdiscus/Fukuyoa* species are also known to produce maitotoxins (MTXs), which are among the largest non-polymeric marine biotoxin molecules known and among the most potent by intraperitoneal injection in mice [29,30]. However, their mechanism of action differs from that of CTXs, and the role of MTXs in human CP remains unclear [20]. 

CTXs are lipophilic, thermostable polycyclic polyether molecules whose neurotoxic effects result from binding to voltage-gated sodium channels (Na_v_) or, to a lesser extent, potassium channels in excitable tissues [31,32,33]. CTXs include numerous analogs of varying potency that have been classified according to their geographical origins, i.e., Pacific (P-CTXs), Caribbean (C-CTXs), and Indian Ocean CTXs (I-CTXs) [34]. Different congeners result from oxidative biotransformation as they are consumed and transferred up the food web [35,36,37]. CTX biotransformation has been largely studied in Pacific marine ecosystems, where toxin profiles have been found to vary among cells, primary consumers, and predators [27,38,39]; yet recent work in the Caribbean to identify the first known algal C-CTX (C-CTX5) has demonstrated that it can be converted in vitro into the C-CTXs commonly found in Caribbean fish (C-CTX1/2 and, to a lesser degree, C-CTX3/4) [37]. In fish, the degree of CTX metabolization in the digestive pathway [40,41] is likely a key factor affecting toxin storage or elimination [20], where increasing polarity with transformation is thought to be a mechanism to facilitate depuration [42,43]. Yet, CTX bioaccumulation, metabolization, and storage in fish can vary considerably among species [44]. Improved understanding of CTX toxicokinetics both within individuals and among trophic levels is key to development of risk assessment and monitoring programs [34,45]. 

Lack of knowledge about the kinetics of CTX (tissue distribution, accumulation, and metabolization) in the entry point to the food web stems from the fact that our understanding of CTX trophic transfer has historically been based on field sampling and toxin assessment of fish and invertebrates of varying trophic levels in the Pacific [27,46,47,48](reviewed by [20,45,49,50]). Some experimental evidence in the last decade, however, has confirmed the rapid absorption and bioaccumulation of CTXs in invertebrates (giant clams) [51,52] and lower-order fish, i.e., mullets [43] and surgeonfish [53], after dietary exposure to *Gambierdiscus*. Recently, conceptual and quantitative models of CTX trophic transfer have been developed that allow the effects of different factors on relative toxicity among trophic levels to be assessed, such as bloom duration and toxin profiles and aspects of fish ecology (e.g., feeding rates and behaviors) [54,55]. The capacity of these models to quantify CTX transfer for risk assessment remains limited by a lack of quantitative data [55,56]. 

In our past work with the acanthurid *Naso brevirostris* [53], we demonstrated that only two weeks’ dietary exposure to toxic *G. polynesiensis* were sufficient to accumulate CTX concentrations in muscle above the threshold for human intoxication. Fish belonging to the family Acanthuridae are primary consumers and considered key vectors of CTXs in the Pacific [54,57]. Species of the genus *Naso* in particular, which are distributed throughout the Indo-Pacific, are often found to contain CTX and have been implicated in CP [47,58]. Recently, aspects of CTX assimilation have been quantified in higher trophic levels by studies in which omnivorous or carnivorous fish were fed with toxic fish flesh (C-CTX: [59,60], P-CTX: [61]) or toxin-infused fish feed (P-CTXs: [62]). Data on the processes of CTX tissue distribution and metabolization in a relevant, known consumer of *Gambierdiscus* spp. cells and vector for CTX trophic transfer into the fish food web are essential for further development of models, yet remain lacking. 

In the context of improved food safety risk management, focusing on the entry point of CTX into the fish food web, we examined the tissue distribution and metabolization of CTX after long-term ingestion of toxic *Gambierdiscus* in an at-risk reef fish. Following the experimental model established in Clausing et al. [53], we exposed juvenile surgeonfish *Naso brevirostris* to cells of a highly toxic strain of *Gambierdiscus polynesiensis* in a gel food diet over 16 weeks. Using two screening bioassays, the Neuro2a cell cytotoxicity assay (CBA-N2a) and radioligand-receptor binding assay (r-RBA), and one confirmatory analytical method by liquid chromatography–tandem mass spectrometry (LC-MS/MS), we quantified toxin content in eight tissue compartments plus the carcass and examined toxin metabolization in the flesh. As *G. polynesiensis* has not been found to produce any known MTXs [63,64], only CTXs were examined.

## 2. Results

### 2.1. Dietary Toxin Exposure 

Over 16 weeks, juveniles of *N. brevirostris* were fed gel food containing cells of *G. polynesiensis* at a rate of 0.36 ng CTX3C equiv g^−1^ fish d^−1^. Cell quantities in food (1333 cells g^−1^) were based on measurement of 4.5 pg CTX3C equiv cell^−1^ in the pellet of harvested cells used for food preparation (CTX activity quantified by r-RBA and confirmed by CBA-N2a at 3.6 pg CTX3C equiv cell^−1^), and dosing rate was confirmed by analysis of the cell-embedded gel food with LC-MS/MS, giving a toxicity of 3.3 pg CTX3C equiv cell^−1^ (full results described previously in Clausing et al. [53]). Throughout dietary exposure, fish consumed *Gambierdiscus*-enriched gel food freely without exhibiting territorial behavior or signs of intoxication (Figure S1). This is consistent with the absence of signs of intoxication in wild-caught ciguatoxic fish [19]. As generally all food was consumed within 60–90 min, we estimate a cumulated dose of 1280.4 ng CTX3C equiv fish^−1^. This is an average corresponding to 5 feedings per week for 16 weeks (78 total exposures) at the estimated dosing rate, where we assume the actual total toxin intake varied among fish according to weight. To maintain a constant rate of toxin consumption over the course of the experiment, the quantity of food given was adjusted for growth, which was estimated from weights of a subset of fish taken biweekly. Growth rate constants were stable over time (Figure S2), validating the use of growth curves constructed periodically from a subset of fish to recalculate quantities of toxic gel food preparation and provide a constant dosing for all individuals. On average, fish increased their body weight by 3.5 ± 0.4% per week (Figure S3), with a total gain of 22.3 ± 7.2 g (final weight of 61.6 ± 16.6 g; Table S1). Although variable, gains tended to be higher in *Gambierdiscus*-exposed fish (26.3 ± 6.3 g) relative to control fish (19.6 ± 8.0 g, all means ± SD).

### 2.2. Toxin Tissue Distribution 

Toxin concentrations in fish tissue extracts were estimated against PbTx3 and CTX3C standard curves using r-RBA and CBA-N2a, respectively (Figure S4). After 16 weeks of CTX exposure, CTXs were measurable in all fish tissues (nine tissues: muscle, liver, spleen, gastro-intestinal (GI) tract, gills, eyes, brain, gall bladder, and carcass) by r-RBA and/or CBA-N2a (Table 1). No detectable amounts of toxin (r-RBA LOD = 0.32 ng CTX3C equiv g^−1^ tissue equivalent (TE) [53]; CBA-N2a LOD = 0.32 pg CTX1B g^−1^ TE [65]) were found in any tissue of control fish (*n* = 3), and no matrix effects were observed. Muscle was the largest of the tissues collected, representing 34–44% of the total fish weight (38.6 ± 3.3% mean ± SD; Table 1). The carcass remaining after tissue collection was similar in weight to the muscle (including bones; 40–50% of body weight, 44.8 ± 3.4 mean ± SD). 

#### 2.2.1. Tissue Concentrations

**Figure 1:**
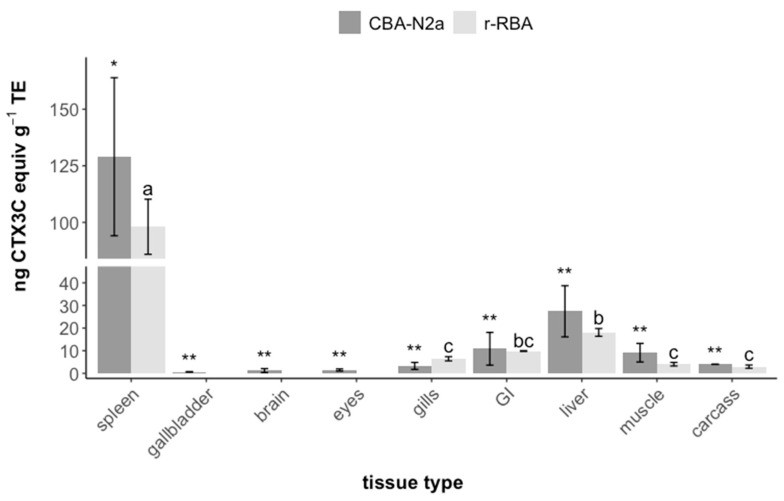
CTX-like activity (ng CTX3C equiv g^−1^ TE) in tissues of *N. brevirostris* after 16 weeks’ dietary exposure to *G. polynesiensis*. All values are given as mean ± SD (*n* = 3–7). Concentrations in all tissues of control fish were below limits of detection (LOD: 0.32 μg CTX3C equiv kg^−1^; *n* = 3). Within each assay, significant differences among tissues (*p* < 0.05, Table 2) are depicted with letters for r-RBA and by asterisks for CBA-N2a. GI = gastro-intestinal tract.

Toxin concentrations varied more than 100-fold among tissues, ranging from less than 1 to more than 100 ng CTX3C equiv g^−1^ TE (Figure 1, Table 1). Concentrations in the spleen were significantly higher than in all other tissues (*p* < 0.05 for both r-RBA and CBA-N2a; Table 2), with concentrations more than 10-fold that of the muscle (5.95 ± 3.48 SD overall, assays combined). Liver concentrations were three to four times those found in muscle and gill tissue and twice that of the gastro-intestinal tract (*p* < 0.05 for r-RBA). The overall trend of accumulation in terms of concentration among fish tissues was as follows: spleen > liver > gastro-intestinal tract > muscle > gills > remaining parts > eyes > brain > gall bladder. Even overall body concentrations (total body burden by total fish mass: 4.5 ± 0.38 and 7.0 ± 2.3 ng CTX3C equiv g^−1^ TE by r-RBA and CBA-N2a, respectively) were orders of magnitude above recommended safety limits, estimated at 0.05 µg CTX3C equiv kg^−1^ (from 0.01 μg CTX1B equiv kg^−1^) [66,67]. Measured toxin concentrations did not significantly differ between the two bioassays (paired *t*-test; t_4_ = 0.19, *p* = 0.857 for a mean difference = 0.437 with CI [−5.86, 6.73]).

#### 2.2.2. Tissue Burden

**Figure 2:**
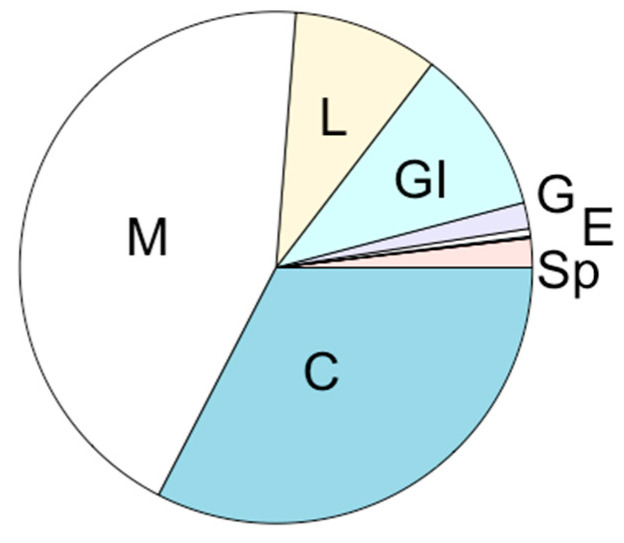
Relative toxin accumulation, i.e., percent of total body burden, in tissues of *N. brevirostris* after 16 weeks’ dietary exposure to *G. polynesiensis*. Values represent means of both screening assays (CBA-N2a and r-RBA). Tissues are labeled as follows: Sp = spleen, E = eyes, G = gills, GI = gastro-intestinal tract, L = liver, M = muscle, C = carcass. The brain and gallbladder are not labeled because the percentage is too small to be seen visually.

Relative tissue distributions calculated from both r-RBA and CBA-N2a data showed that ciguatoxin activity was mainly retained in muscle and the carcass (~45% and ~33%, respectively; Table 3, Figure 2), the largest tissues (Table 1). Relatively high CTX accumulation levels were also recorded in the gastro-intestinal tract (~10%), with similar quantities of CTX in the liver (~9%) (Figure 2), despite its smaller size (~3-fold smaller). With a mass 20–100-fold less than other viscera, the spleen represented only ~2% of the total body burden, even with its elevated CTX concentration. Mean total body burden was estimated to be 283.8 ± 64.5 and 385.8 ± 120.8 ng CTX3C equiv by r-RBA and CBA-N2a, respectively. As carcass samples were not directly analyzed by CBA-N2a, the total burden was calculated using an estimated concentration based on r-RBA toxin proportions between the carcass and other tissues (Table S3). The calculated tissue burden for r-RBA lacks the brain, eyes, and gallbladder. However, these tissues made up only 0.61% together of the total body burden by CBA-N2a, so their absence is not expected to significantly change the estimated body burden by r-RBA. Some variability in total toxin burden is expected due to differences in body mass among fish and, presumably, proportionally higher rates of consumption. Total tissue burden showed an increasing, yet saturating trend with body weight (Figure S5; Table S4). 

**Figure 3:**
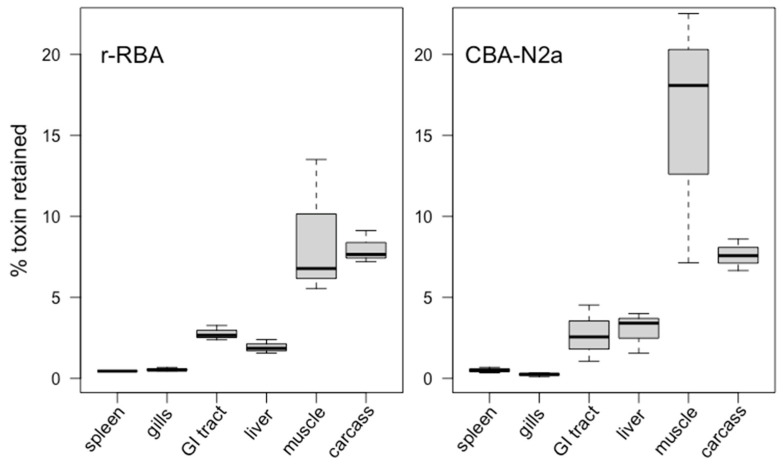
Percent of total dose of toxin retained in each tissue type as determined by r-RBA (*n* = 3–7) and CBA-N2a (*n* = 3).

The percentage of total toxin intake after 16 weeks’ dietary exposure that was assimilated in fish tissues was estimated at 22.2 ± 5.0% by r-RBA and 30.1 ± 9.4% by CBA-N2a (Table 4). The relative toxin retention among tissues was proportional to trends of tissue burden, with most of the ingested toxins accumulated in the flesh (~12%), followed by the carcass (~8%; Figure 3). There were no significant differences in percent CTX accumulation between methods (paired *t*-test: t_5_ = 0.32, *p* = 0.76 for a mean difference = 0.12 with CI [−0.88, 1.13]). Higher apparent CTX retention in muscle by CBA-N2a (Figure 3) likely results from true variability among fish, where different subsets of fish were quantified by each method (selected randomly, with overlap). 

Compared with our previous *Gambierdiscus*-feeding study with *N. brevirostris* [53], fish retained a significantly higher percentage of ingested CTXs in their flesh (*t*-test: t_4.7_ = −3.43 and *p* = 0.025 with means of 2.05% and 13.3%, respectively). Experimental fish in Clausing et al. [53] received the same food at the same rate of daily dosing but were of smaller weight (*t*-test: t_6.3_ = −4.67 and *p* = 0.003 with initial mean biomass of 6.57 g vs. 39.6 g, respectively). These smaller fish in the previous study also grew faster, with 325% growth from ~7 to ~28 g [53] compared with 72.5% mean increase from ~40 to ~62 g in the present study (*t*-test: t_4.7_ = 8.64 and *p* < 0.001).

### 2.3. Toxin Metabolization

**Figure 4:**
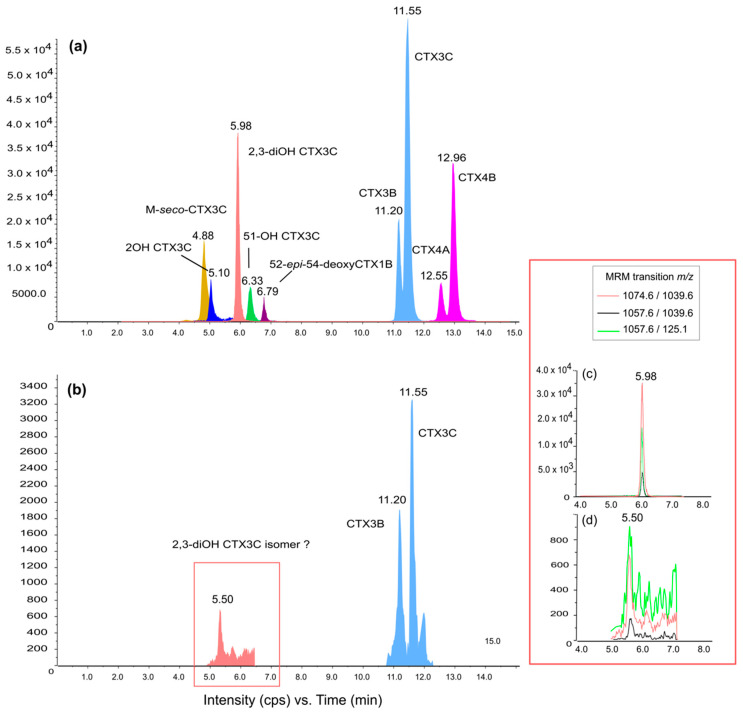
Total Ion Chromatograms (TIC) acquired in positive multiple reaction monitoring (MRM) mode representing (**a**) a mixture of Pacific ciguatoxin standards and (**b**) the toxin profile of a muscle extract of *N. brevirostris* after 16 weeks’ feeding with *G. polynesiensis*. Extract Ions Chromatograms (EIC) of (**c**) the *m*/*z* transitions of 2,3-dihydroxyCTX3C standard and (**d**) the corresponding peak of interest at 5.50 min from the muscle extract.

LC-MS/MS analysis of *N. brevirostris* fed with *G. polynesiensis* showed detectable levels of CTXs accumulated in the muscle tissue in two of the three fish analyzed (Figure 4; limit of detection (LOD) = 0.05 ng CTX3C equiv g^−1^ TE). Retention times and characteristic ion ratios of the CTX standards (Figure 4a) were consistent with those of the sample extracts, revealing the presence of CTX3C and CTX3B (1.08 and 0.79 µg CTX3C equiv kg^−1^, respectively; Figure 4b). This follows from the toxin profile in the gel food, which consisted of predominately CTX3C, followed by CTX3B (LC-MS/MS analysis of gel food reported in Clausing et al. [53]). Furthermore, a compound corresponding to the three multiple reaction monitoring (MRM) transitions of 2,3-dihydroxyCTX3C was also detected at 5.508 min (0.19 µg CTX3C equiv kg^−1^), but with a different retention time than the standard (5.980 min) and a different ion ratio (Figure 4c,d). This compound appears to be an oxidation product of CTX3C or CTX3B resulting from fish metabolism.

## 3. Discussion

Fish are well documented as a primary vector for human ciguatera. In this study, the route of CTX exposure in an herbivorous, CP-prone fish was replicated ex situ by feeding juvenile *Naso brevirostris* with gel food containing toxic *Gambierdiscus polynesiensis* cells under controlled laboratory conditions, allowing for assessment of toxin transformation and accumulation among tissues. We evaluated toxin transformation by LC-MS/MS analysis of CTX analogs in the flesh and estimated CTX activity among different tissues using r-RBA and CBA-N2a, which allowed both direct comparison with our previous study [53] and quantification of small tissue samples. The general estimates and relative distribution of CTX activity among tissues differed neither qualitatively nor statistically between the screening assays and are thus discussed as averages. All interpretation hereafter is made acknowledging the limitations inherent in the methods used for CTX determination as well as the constraints associated with our experimental in vivo exposure using an animal model, which include use of minimal numbers of juvenile organisms, with resulting limits on sample availability and replication. 

### 3.1. Toxin Tissue Distribution 

Experimental dietary exposure to *G. polynesiensis* resulted in the detection of significant, but highly variable concentrations of Pacific CTXs in all analyzed tissues of *N. brevirostris*, indicating that these toxins are distributed throughout the body. Lower-order fish belonging to the Acanthuridae are considered key vectors of CTXs in the Pacific [54,57], and *Naso* spp. are often considered unsafe for human consumption [57,68]. Apart from reports of CP cases, evidence is based primarily on CTX analysis of the flesh [27,47] and secondarily the liver [46] of field-collected specimens. To understand the kinetics of toxin assimilation and elimination at this first level of exposure, quantification of CTX distribution among tissues is essential, yet such studies remain scarce and limited to higher-trophic level fish or fish models [43,59,62,69]. To our knowledge, this is the first study to experimentally demonstrate that consumption of toxic *Gambierdiscus* results in accumulation of significant CTX concentrations in all major organs of a relevant herbivorous fish and known vector of CTXs into the fish food web.

Sustained exposure to toxic *Gambierdiscus* resulted in the highest concentrations of CTXs in the spleen and the liver, at approximately 110 and 20 ng CTX3C equiv g^−1^ TE (combined r-RBA and CBA-N2a data). Flesh concentrations were at least 10-fold and 3-fold lower than those of the spleen and liver, falling in range with levels in the gills and gastro-intestinal tract at 5–10 ng CTX3C equiv g^−1^ TE. These results are in general agreement with previously reported concentrations in fish flesh contaminated by CTXs, as determined by r-RBA and/or CBA-N2a [53,57,68,70], including those of field-collected *N. brevirostris* (1–7 ng CTX3C equiv g^−1^ TE) [47]. High CTX concentrations in the liver and viscera relative to the flesh have also been found in other studies of both field-collected and experimentally fed fish [46,59,69,71,72], although at widely variable ratios (e.g., liver: 3–100 and viscera: 1–14-fold [69]; liver: 3–69 and viscera: 42–330-fold [59]; liver: 1–100+ fold [72]). In our study, measured CTX concentrations in visceral organs were variable, yet the ratios with muscle concentrations were relatively stable for both liver (4.00 ± 0.94, *n* = 5) and the GI tract (1.76 ± 0.78, *n* = 5). This reduced variability compared with other studies may result from differences in the toxin source (cells in this study vs. toxic fish flesh in the others) and level of exposure (10-fold higher dose [59]; unknown exposure of field-collected fish [69,71,72]), or in the physiology and processes of metabolization of the consuming fish (herbivores vs. omnivores or carnivores). In field-sampled fish, it may also relate to variability in the timing and extent of exposure, as CTXs redistribute among tissues over time post-consumption [43,59]. 

The highly variable CTX concentrations observed among tissues may provide insight into the relative roles of these organs in toxicokinetic processes. The spleen and the liver are highly vascularized organs that serve as common sites for blood storage and sequestration of harmful compounds and contaminants such as heavy metals (e.g., lake fish [73,74]); thus, these organs may receive significant CTX inputs through blood circulation. Additionally, CTXs have been found to show affinity to cytoplasmic proteins in the liver (C-CTX: [69]), which is involved in protein synthesis. The spleen, in turn, may play a role in fish immune responses (secondary to the head kidney), where lymphocytes and macrophages have been found to intercept toxins (PbTx3), although direct evidence for CTXs is lacking (reviewed by [75]). 

High CTX concentrations in the gastro-intestinal tract may be due to recent consumption and absorption by enterocytes on the luminal surface of the intestine, which is the first step of xenobiotic metabolism. Conversely, high levels may be due to enterohepatic recirculation, as fish were sacrificed 24 h after the last toxic meal. This corresponds to the finding of Ledreux et al. [43] that intestines of mullet contained an increasing proportion of consumed CTX over 24 h after a single feeding with *G. polynesiensis.* Another study on carnivorous grouper found that the proportion of toxin retained in the intestine increased as that of the liver decreased in the 15 days following extended exposure [62]. This suggests that enterohepatic recirculation or other means of redistribution may also continue to augment proportional toxin retention in the intestine beyond 24 h. 

The eyes are not known to be involved in CTX depuration, but have recently been shown to accumulate high concentrations (fourfold those in muscle tissue in grouper [76]). We found significant, but lower concentrations (sixfold lower than flesh), providing evidence that the eyes may serve as a storage site for CTXs in both herbivorous and carnivorous fish.

The presence of CTX activity in the gills may indicate that the respiratory pathway is involved in toxin processing and excretion, as has been suggested for mullet [43] and grouper [62]. CTX excretion in bile following hepatic metabolism is also considered a potential route for rapid depuration, where mullet gallbladders contained 10% of the total CTX burden 24 h after exposure [43]. In contrast, *N. brevirostris* gallbladders retained low levels of CTXs. This provides evidence that processes of CTX metabolism, assimilation, and elimination may be species-specific, or at least vary temporally post-CTX consumption. Our results provide a snapshot after 16 weeks’ daily exposure; further work including tissue collection over short (hours) and long (days/weeks) temporal scales post-exposure is needed to understand the dynamics of transitory vs. longer-term CTX storage.

### 3.2. Toxin Metabolization

The metabolism of CTXs within the fish was investigated using LC-MS/MS to search for biotransformed products. Metabolism is believed to play a key role in toxin storage and depuration in fish. It has been suggested that oxidative transformation of consumed CTXs into more polar analogs may be a strategy to facilitate toxin elimination [20,42]. Despite the prevalence of oxidized congeners in upper trophic levels [27,38], acidic digestive tracts in some herbivorous fish may catalyze the production of polar CTX analogs through (acid-catalyzed) spiro-isomerisation [20,41]. Accordingly, CTX profiles in Pacific acanthurids have been found to contain more metabolized analogs, including CTX4B [77], the biotransformation intermediates 52-*epi*-54-deoxyCTX1B and 54-deoxyCTX1B, and lesser amounts of the biotransformation end-product, CTX1B [27]. 

In concordance with the CTX profiles previously determined for the gel food [53] and for the TB92 strain of *G. polynesiensis* used in the gel food preparation [64,78], LC-MS/MS analysis confirmed the presence of CTX3C and CTX3B in *N. brevirostris* muscle extracts. The analysis of the toxic fraction led also to the detection of a chromatographic peak at 5.50 min indicating the *m*/*z* transitions of the 2,3-dihydroxyCTX3C. The *G. polynesiensis* strain used in our experiment (TB92) has been shown to contain CTX3C, CTX3B (49-epiCTX3C), CTX4A, and, in small quantities, CTX4B, M-seco-CTX3C, 2-OH-CTX3C and four isomers of CTX3C [53,64,78,79]; 2,3-dihydroxyCTX3C has not been detected in TB92 or other strains of *G. polynesiensis* [63]. Although one study found that 2,3-dihydroxyCTX3C is a main component of the toxin profile of some Vietnamese strains of *G. toxicus* [80], this has been suggested to result from a misidentification of the parent ion of a gambierone group analog commonly found in *Gambierdiscus* [81]. Thus, the presence of the biotransformed CTX analog 2,3-dihydroxyCTX3C in the muscle of *N. brevirostris* provides evidence for bio-oxidization of algal ciguatoxins as they move up the food web [82]. Ikehara et al. [36] demonstrated oxidative conversion of *Gambierdiscus* CTXs (CTX4A/4B and CTX3C) in vitro in liver extracts (S9 fractions) from carnivorous and omnivorous fish known to be ciguateric. One of the products of CTX3C incubation with *Lutjanus bohar* and *Oplegnathus punctatus* livers was 2,3-dihydroxyCTX3C. Our results indicate that a common mechanism for enzymatic biotransformation by hepatic metabolism may also exist in herbivorous fish. Interestingly, Ikehara et al. [36] also found that incubation of CTX4A/4B with livers of *Lutjanus* species not implicated in CP resulted in very little production of known oxidative metabolites. This suggests that even within genera, xenobiotic metabolism may differ among species, with different oxidation products and potentially different associated risks for ciguatoxicity. Studies are needed to elucidate the role of metabolization in CTX bioaccumulation, elimination, and toxicity in fish and how it varies with trophic level, geographical origin, and species of *Gambierdiscus* consumed [83].

### 3.3. Toxin Tissue Burden 

While tissue CTX concentrations indicate toxin distribution within individual fish organisms, assessing tissue burden is valuable for evaluating the potential for trophic transfer within the food web [55]. Despite high CTX concentrations in the viscera, the largest proportion of CTXs retained was in muscle tissue and secondarily the carcass (~45% and ~35% of total body burden, respectively), which likely reflects storage mechanisms as well as the relatively high total mass of these tissues compared with other organs (~40 and 45% of total body mass, respectively, representing ~20× liver and ~500× spleen masses). Toxin retention in fish heads, skin, and bones is generally considered low [69,84] and disregarded in studies of CTX tissue distribution [43,59,62,71] (see Vernoux et al. [69] for an exception). While fish eyes have been shown to accumulate significant toxin concentrations [84]), their size likely renders the contribution to tissue burden negligible (0.48% of total body burden in the present study). Although we cannot confirm the presence of toxin in each individual part of the carcass of *N. brevirostris* (head minus eyes, bones, fins, cartilage, and skin) without explicit analysis, its significant overall CTX burden suggests that the carcass should not be neglected and may represent a significant compartment of bioaccumulation from which toxin may be redistributed to other tissues over time.

Despite the accumulation of ~300 ng CTX3C equiv in the tissues of ~70 g *N. brevirostris* over 16 weeks of daily feeding on toxic *Gambierdiscus* (0.36 ng CTX3C equiv g^−1^ fish d^−1^), these herbivorous fish continued to consume toxic food readily with no obvious signs of intoxication. Yet, omnivorous and carnivorous fish have shown responses to toxic food even at significantly lower doses. A study on lionfish exposed to toxic parrotfish flesh (2.3 ng CTX3C equiv g^−1^ TE) at a dose of 0.1 ng CTX3C equiv g^−1^ fish d^−1^ (0.035 ng by CBA-N2a) showed feeding inhibition after 5 weeks’ exposure [61]. Goldfish consuming 0.014 ng CTX1B equiv (~0.075 ng CTX3C equiv) g^−1^ fish d^−1^ daily showed severe symptoms while feeding by the 6th day [60]. Striped mullet given a similar dose (0.3 ng CTX3C equiv g^−1^ fish d^−1^) were evidently intoxicated by the second consecutive feeding [43]. Among these species, lionfish, mullet, and unicorn fish have commercial value and could pose a risk to public health in CP endemic areas; goldfish are not naturally exposed to CTXs. Worthy of note, unicorn fish (e.g., *N. brevirostris*) are regarded as relevant sentinel species in CP risk surveillance programs, such as those currently conducted in French Polynesia [39]. Furthermore, these varied results highlight the differences in CTX tolerance among fish species [16] and suggest that a resistance mechanism may have evolved in *N. brevirostris* that alters receptor sensitivity or reduces CTX bioavailability. A pathway for toxin resistance has been considered for saxitoxins and tetrodotoxins (two potent neurotoxins also binding to the Na+ channels) involving toxin-binding protein in the liver, intestines, ovaries, and skin of poisoned pufferfish [85]. In liver cells, the spontaneous formation of the ciguatoxin protein complexes was observed in vitro [86]; however, this requires further investigation. 

After 16 weeks’ exposure, 26% of total ingested toxin (1280 ng fish^−1^ on average) was assimilated in fish tissues. A study feeding omnivorous pinfish with toxic flesh of field-collected barracuda found that pinfish retained 43% of consumed C-CTX1 over 20 days [59]. Although they are known to have similar backbone structures, it is unknown how the toxicokinetics may vary among the well-characterized Pacific CTXs and the much lesser known Caribbean CTXs [87]. Additionally, the toxin CTX profile of carnivorous fish contains metabolized and generally more oxidized congeners than that of *Gambierdiscus* [20,39], which may be assimilated at different efficiencies. For example, grouper have been shown to assimilate CTX1, 2, and 3 at different rates [62]. Moreover, toxin exposure was nearly 20-fold less (0.02 vs. 0.36 ng CTX3C equiv g^−1^ fish d^−1^), and accordingly, so were body burdens (15 vs. 300 ng CTX3C equiv), suggesting that the quantity retained may be proportional to that consumed. A study on striped mullet from the southeast USA, in contrast, found that fish fed with a similar dose of *G. polynesiensis* (0.3 ng CTX3C equiv g^−1^ fish) eliminated 99% of toxin within 8 h after nine feedings over 16 days [43]. These varied results demonstrate how depuration may vary among species and with toxin profile [54]. Overall, the high percentage of toxin retained over months of repeated dietary exposure signifies that *N. brevirostris* have a high capacity to accumulate toxin and suggests that elimination of CTXs may be a slow process (see also [26]).

### 3.4. Implications for Trophic Transfer and Seafood Safety 

Standards for seafood safety focus on toxin content of the flesh as the dominant part of the fish consumed (although in some Pacific islands, other organs may be commonly consumed) [88]. Compared with our previous work with *N. brevirostris*, CTX concentrations in the flesh were similar (means of ~3 and ~4 ng CTX3C equiv g^−1^ TE in the past [53] vs. current study, respectively); however, the percent of ingested CTXs retained was approximately 6-fold higher (10–15%) than that found previously in the flesh of smaller fish (2%). It is possible that this higher relative assimilation relates to the lower growth rates observed in larger fish in the present study (6-fold larger at the experimental onset with 4.5-fold lower growth over the experimental duration). One explanation may be that faster-growing fish metabolize xenobiotics more rapidly, where conversion into more polar compounds may render CTX excretion more efficient [20,42]. Yet, within the size range of our experimental fish, total tissue burden appeared to be saturating in the largest fish. Although this trend is preliminary, being based on a very limited sample size, this may suggest that the rate of depuration is concentration-dependent in this fish species. Alternatively, increasing toxin accumulation may shift metabolization to analogs with lower Na_v_ affinity, reducing estimates of toxicity by bioassay methods. Finally, age- or size-specific changes in feeding habits or physiological factors may also alter toxin uptake or kinetics as fish grow, although in the field such patterns may be overridden by species- or site-specific factors [47,58]. Overall, our study does not allow extrapolation to adult fish, but does suggest that slower growth rates in juvenile fish may be associated with increased accumulation of ingested toxin in muscle tissue. As toxin tissue distribution was not examined in Clausing et al. [53], however, we cannot be certain that differences relate to elimination; in the future, toxin measurements of the feces of exposed fish could provide a means to differentiate between excretion and redistribution to other tissues.

The concentrations observed in these small fish far exceed the recommended safety limits for human consumption (0.05 μg CTX3C equiv kg^−1^) [66,67]. Following that rates of growth continue to decline as fish increase in size, somatic growth dilution may not be sufficient to reduce CTXs to safe concentrations, as has been shown in recent models [54]. Moreover, Holmes and Lewis [56] suggest that turf-eating acanthurids may be consuming significantly higher quantities of cells per day than our rates of exposure (89 cells g^−1^ fish d^−1^). Increased cell consumption would amplify exposure, potentially magnifying toxin accumulation throughout the animal. While our fish showed no signs of intoxication or other behavioral effects of repeated CTX consumption, it may be possible that a species-specific threshold of toxin exposure exists, after which fish may exhibit health problems [60] or be deterred from eating [e.g., [61]]). Similarly, as discussed above, it is unknown how the balance of toxin assimilation and elimination may change under continued exposure as fish become larger, growth rates slow, and CTXs accumulate in fish tissues. Size-related changes in the balance between ingestion and excretion have important implications for seafood safety, particularly if larger fish targeted by fisheries retain a higher proportion of ingested toxin as body burden. Continued feeding studies including larger fish and varying exposure rates are needed to complement field collections toward clarification of predictive models for CTX trophic transfer and risk assessment. Such models are essential to improving seafood safety as the distribution of CTX-producing dinoflagellates continues to widen, with accompanied occurrences of seafood poisoning.

## 4. Materials and Methods

### 4.1. Experimental Model 

#### 4.1.1. Study Species

To examine the fate of CTX in a relevant first-order consumer after long-term dietary exposure, we fed juvenile *N. brevirostris* with intact *G. polynesiensis* embedded in a gelatin-based food in a controlled laboratory setting over 16 wks. Highly toxic cells of *Gambierdiscus polynesiensis* from the strain TB92 (Tubuai, Australes archipelago, French Polynesia) were obtained from the Institut Louis Malardé (French Polynesia, culture and harvest conditions as described in Clausing et al. [53] and Chinain et al. [78,89]). Toxin concentrations estimated in TB92 range from 3.4–11.9 pg CTX3C equiv cell^−1^ (by r-RBA, CBA-N2a) [51,53,78], depending on the growth phase (higher cell toxin content in the stationary phase) [see also [63,78]]). Pelleted cells were stored at −20 °C until use. 

Wild-caught juvenile *Naso brevirostris* (Acanthuridae), a common species of surgeonfish throughout coral reefs in the Indo-Pacific, were obtained from the Maldives through a fish wholesaler (Tropic Nguyen, Kingersheim, France). Juvenile *N. brevirostris* are herbivores, feeding on benthic macroalgae, while adults are mostly planktivorous. Fish were caught on the reef and acclimatized 15 days before shipment to the International Atomic Energy Agency Marine Environment Laboratories (IAEA-EL) in Monaco (information provided by the supplier). *N. brevirostris* were chosen as the model fish species because they are known to be involved in human CP and CTXs have been detected in the flesh of wild-caught specimens [47] and after trophic transfer from cells in the laboratory [53].

#### 4.1.2. Fish Maintenance

Fish were acclimated to laboratory conditions for 3 months prior to dietary exposure, first in an open-circuit 500 L tank (100 L h^−1^ of 0.45 μm filtered seawater maintained at 25 ± 0.5 °C and 38 psu with a 12 h light: 12 h dark cycle) for 2 months, and then 1 month in pairs in individual 100 L tanks that also served as experimental units (identical conditions). Fish were fed a regimen of 10% body weight d^−1^ consisting initially of dried algae (Green Marine Algae, Ocean Nutrition^TM^, Essen, Belgium) and brine shrimp (*Artemia salina*) with gradual incorporation of gel food (GellyBelly^TM^ Gel Food, Florida Aqua Farms, Inc., Dade City, FL, USA, preparation detailed below) composed of microalgae, macroalgae, fish and krill meal, and vitamins and minerals mixed with gelatin. Throughout the experiment, handling was minimal to avoid physiological stress, and standards of animal welfare were carefully maintained following international guidelines (EU regulations on animal research: https://www.eara.eu/animal-research-law, (accessed on 26 October 2023)).

#### 4.1.3. Gel Food Preparation

Gel food for both control and exposure treatments was prepared following Clausing et al. [53]. Briefly, gel food was prepared from GellyBelly^TM^ dry powder and *G. polynesiensis* cells homogenized in 75 °C seawater (control food without cells) at a ratio of 0.67 g powder mL^−1^ seawater. Light microscopy confirmed that frozen cells were intact before use and that seawater temperature had no obvious effect on the integrity of the cells (i.e., lysis). Both control and dosed food were mixed manually in plastic bags (>3 min) to ensure homogenization and aliquoted onto nylon screen (2 mm mesh size). Gel food was stored at −18 °C until feeding (minimum 10 min to solidify). The final concentration of *G. polynesiensis* was 1333 cells g^−1^ gel food, where the total quantity of food given was adjusted as the fish grew but the concentration of cells remained constant. Laboratory tests verified that no cells or CTX leached from the food into the water over a period exceeding the duration of feeding. These tests, including microscopic observation of cells and CTX measurements at 1 h intervals in water in which gel food was soaked for 3 h (3 g in 5 mL; methods and results described in Clausing et al. [53]), confirmed the stability of the gel food preparation and diet as the sole means of exposure. The cells used for the gel food were from the same harvest of TB92 culture as in Clausing et al. [53].

#### 4.1.4. Fish Exposure

After the initial acclimation period, juvenile *N. brevirostris* (ranging 23.37–47.09 g, *n* = 12) were distributed equally according to size among six 100 L aquaria (conditions described above; *n* = 2; 69.8 ± 2.3 g mean ± SE total biomass per tank). To avoid stress, aquaria walls were covered with opaque plastic and fish were provided with PVC tubes as habitat (three 10 cm × 20 cm D × L tubes stacked into a pyramid). 

After random assignment of aquaria to exposure or control treatments (*n* = 3), biomasses of control and experimental fish were 37.0 ± 9.5 and 32.8 ± 5.3 g, respectively (mean ± SD, *n* = 6). Over 16 weeks, fish were fed gel food five days per week in the morning at a constant proportion of 6% of their biomass. Fish in exposure tanks were given gel food containing *G. polynesiensis* to achieve a dose of 89 cells g^−1^ body weight d^−1^. Control fish consumed gel food without cells. Given the proportion of cells to gel food (0.00013 g cells g^−1^ gel food), it was deemed unnecessary to account for the presence of cells in the gel food of control fish. Cell consumption rates were based on estimated *Gambierdiscus* spp. bloom densities (see discussion in [53,90,91]) and the quantity of food required by juvenile fish (5–10% body weight d^−1^). Food quantities were adjusted for growth every 1–2 weeks, where fish from one control and one experimental tank were weighed in rotation every second week to avoid unequal stress among treatments or replicate tanks. All fish were weighed four times in total. Growth was estimated from measured increases in biomass and extrapolated to all fish using the exponential growth equation. Fish were fed simultaneously and observed during the feeding period to confirm that both fish in each tank ate. Over 16 weeks, fish received 78 feedings (two days were missed due to laboratory closure). Brine shrimp were provided in the afternoon as a food complement to reach a daily ration of 10% body weight d^−1^. 

Twenty-four hours after the last feeding, fish were euthanized with eugenol (0.4 mL L^−1^; induction time < 10 s). Dissection was performed immediately after euthanasia was confirmed by the absence of gill movement (~10–12 min). Fish were sacrificed one at a time to minimize the delay before sample collection. Samples were divided by entire tissues, including muscle, liver, spleen, gastro-intestinal tract, gills, eyes, brain, and gall bladder; all tissue types were isolated from each fish. Tissue samples were placed in pre-weighed vials, weighed, and immediately frozen and stored at −20 °C until analysis. Weight among tissues varied a magnitude of order from tens of milligrams to tens of grams. All remaining parts (head, bones, skin, fins, and spines) were combined into a composite sample for each individual, hereafter termed the carcass. Weighed carcass samples were lyophilized (Labconco FreeZone 12 L freeze dry system, Kansas City, MI, USA), ground into powder, and stored at room temperature. Exposed and control fish were sacrificed and processed in a random order in an identical fashion. 

### 4.2. Toxin Determination 

#### 4.2.1. Sample Extraction

Toxin content of *G. polynesiensis* was determined by radioligand-receptor binding assay (r-RBA) and a neuro2a cell-based assay (CBA-N2a) after cell extraction according to Chinain et al. [90] with modification as described in Clausing et al. [53]. Gel-food containing *G. polynesiensis* was analyzed by liquid chromatography–tandem mass spectrometry (LC-MS/MS; data previously published in Clausing et al. [53]).

Lyophilized carcasses were reconstituted to original wet weight with Milli-Q. Frozen fish tissues and reconstituted lyophilized carcasses were extracted following Lewis et al. [40] with minor modifications (described in [53,92]). Briefly, cooked samples (15 min in 70 °C water bath) were homogenized with a T-25 digital Ultra-Turrax (IKA Works, Staufen, Germany) and extracted in acetone (3 mL g^−1^ sample) with probe sonication (2 min) and centrifugation. Lipophilic compounds were removed by solvent–solvent separation of 90% aqueous methanol (aq MeOH) with n-hexane. Samples were then diluted to 60% aq MeOH, and CTXs were isolated by solvent–solvent separation with dichloromethane (DCM). The CTX-containing organic phase was evaporated under nitrogen (TurboVap LV Evaporator, Biotage, Uppsala, Sweden), resuspended in 100% MeOH, and stored in glass vials at −18 °C until toxin analysis.

#### 4.2.2. Radioligand-Receptor Binding Assay (r-RBA)

CTX concentrations in *G. polynesiensis* cells and fish tissue extracts were quantified using a radioligand-receptor binding assay (r-RBA) [93] in microplate format (minor modification sensu [92,94]). In the r-RBA, CTXs in the sample extract competitively displace a known quantity of tritiated brevetoxin ([^3^H]PbTx3) at their shared receptor on the voltage-gated sodium channel [82,95], allowing quantification by the reduction in beta emissions. Reagents included phosphate buffered saline buffer (PBSTTween^®^) with bovine serum albumin (1 mg mL^−1^), a [^3^H]PbTx3 working solution (1 nM assay concentration; Latoxan, Portes-lès-Valence, France), and a dilute membrane preparation of porcine brain homogenate (0.8 mg protein mL^−1^; Sigma-Aldrich, St. Louis, MO, USA). After preparation with CTX3C standard (2.85 × 10^−9^ to 1.92 × 10^−12^ M; Wako-Pure Chemicals, Osaka, Japan) and sample extracts, microplates were incubated for 1 h at 4 °C and filtered before 2 h dark incubation with liquid scintillant (Optiphase, PerkinElmer, Hopkinton, MA, USA) and beta counting (MicroBetaPlate Counter, PerkinElmer). Toxin levels in samples were estimated against CTX3C standard curves (present on each microplate) and expressed as CTX3C equivalents (equiv). Tissues were analyzed based on tissue extract availability and included muscle, liver, spleen, GI tract, gills, and the carcass (i.e., all remaining parts; *n* = 3–7). The smallest tissues (brain, eyes, and gall bladder) could not be run due to tissue mass available (ranging 0.07 to 1.22 g), where preference was given to analyze individual, replicate samples by CBA-N2a rather than pooling samples within treatment in order to analyze one composite sample for each tissue type by r-RBA. Sample dilutions were run in triplicate, retaining only those that fell within the linear portion of the standard curve and had an acceptable RSD (<30% among triplicate counts). The RSD also assessed intra-assay variability, while inter-assay performance, variability, and acceptability were determined by a PbTx3 QC (<30% true value) and assay standard curve parameters (EC50, slope, and 100% binding). All samples were analyzed in 2–4 independent r-RBA assays. CVs among assays ranged 0.6–24.6% with a mean of 8.3.

#### 4.2.3. Neuro2a Cytotoxicity Assay (CBA-N2a)

Ciguatoxin activity in *G. polynesiensis* extracts and fish tissue extracts was also analyzed using a cell-based assay, the Neuro2a cytotoxicity assay (CBA-N2a) [96]. The CBA-N2a is a ouabain/veratridine-dependent in vitro screening method that provides a sensitive measure of overall toxicity based on CTX properties that activate the voltage-gated sodium channel (VGSCs) [97]. Its high sensitivity requires little sample material [98] and allowed us to analyze all eight sampled tissues (excluding the carcass; *n* = 3), including the spleen (~50 mg), gall bladder (90 mg), and brain (~250 mg) of juvenile fish. 

The mouse neuroblastoma-2a cell line (CBA-N2a) was obtained from the American Type Culture Collection (ATCC CCL 131) and grown in culture flasks in RPMI 1640 medium supplemented with 10% fetal bovine serum (FBS) and 1mM sodium pyruvate. Cells were maintained at 37 °C in humidified 5% CO_2_ atmosphere. For experiments, CBA-N2a cells were harvested with a trypsin-EDTA solution and plated in a 96-well microplate in 5% FBS RPMI (100 µL well^−1^; Costar, Cambridge, MA, USA) at a density of 30,000 cells well^−1^. Plates were incubated overnight to allow cells to adhere and cover ~70% of the well surface. 

CTX3C standards (Wako-Pure Chemicals, Osaka, Japan) or extracts (*G. polynesiensis* cells or fish tissue, resuspended in MeOH) were added (1 μL) to Ouabain (0.5 mM)/Veratridine (0.05 mM)-pretreated CBA-N2a cells. Controls lacking O/V pretreatment were also used to quantify toxic effects potentially caused by non-sodium channel binding compounds present in the extracts. 

After 18–24 h of exposure, cell viability was assessed using the colorimetric MTT test, i.e., the 3-(4,5-dimethylthiazol-2-yl)-2,5-diphenyl tetrazolium bromide assay [99]. After removing the incubation medium, 50 µL MTT tetrazolium salt in 5% RPMI was added to each well, and the microplates were incubated for 2 h. MTT is reduced by the mitochondria of metabolically active cells into a blue formazan product. The MTT was discarded, and 100 µL dimethyl sulfoxide was added to each well to dissolve the formazan. Absorbance was then measured at a wavelength of 550 nm using a 96-well plate reader (BioTek Winooski, VT, USA, Epoch 2). Assays were repeated three times for each sample, with each sample run in duplicate in the plate.

#### 4.2.4. Liquid Chromatography–Tandem Mass Spectrometry (LC-MS/MS) 

LC-MS/MS analyses of P-CTX were performed only on muscle extracts of exposed fish (*n* = 3). The dried muscle extracts were resuspended with MeOH 100% prior to LC-MS/MS analysis. The quantitative targeted analysis of CTXs was investigated according to Sibat et al. [79]. Qualitative assessment of the CTX profiles was carried out using an LC system (UFLC Nexera, SHIMADZU, Kyoto, Japan) coupled to a hybrid triple quadrupole/ion-trap API4000 Qtrap mass spectrometer (Sciex, Framingham, MA, USA) equipped with a turbo spray^®^ interface. A C_18_ Zorbax column (Agilent technologies, Santa Clara, CA, USA) was employed with a linear gradient using water as eluent A and MeOH as eluent B, both eluents containing 2 mM ammonium formate and 50 mM formic acid. The flow rate was of 0.4 mL min^−1^, the injection volume was 5 µL, and the column temperature 40 °C. The elution gradient was as follows: 78% B to 88% B from 0 to 10 min, hold at 88% B for 4 min, decrease from 88% to 78% in 1 min, and hold for 5 min at 78% B.

The instrument control, data processing, and analysis were conducted using Analyst software 1.7.2 (Sciex Framingham, Framingham, MA, USA). Mass spectrometry detection was performed in positive mode using scheduled Multi Reaction Monitoring (MRM) scanning two or three transitions for each toxin. The selected *m*/*z* transitions are listed in Table S5. Calibration solution of CTX3C (Wako-Pure Chemicals, Osaka, Japan) was prepared in MeOH with concentration ranging from 6 to 400 ng mL^−1^. In addition, a mix of P-CTX standards (ILM, Papeete, French Polynesia) was injected to obtain a reference for the retention times (Figure 4a).

### 4.3. Data Analysis 

r-RBA and CBA-N2a sample toxin data were determined by fitting 4 parameter-sigmoidal standard curves with variable Hill slope using GraphPad Prism version 6.0 (San Diego, CA, USA). Toxicity was expressed as pg CTX3C equiv cell^-1^ of *G. polynesiensis* and as ng CTX3C equiv g^-1^ fish tissue equivalent (TE). Differences in the methods of determination were evaluated by comparing a) toxin concentrations and b) proportion of ingested toxin retained as measured for each tissue type (muscle, liver, spleen, GI tract, and gills) using paired *t*-tests. For each bioassay, toxin concentrations among tissues were compared with ANOVA followed by Tukey’s HSD test. As carcass samples were not analyzed by CBA-N2a due to logistical constraints, toxin concentrations by CBA-N2a in this tissue type were estimated using the proportion of r-RBA-measured CTX3C in the carcass relative to other tissues (*n* = 5; details of calculation in Table S3). The resulting CBA-N2a carcass estimates given by each tissue type were averaged to give an approximate value, allowing for a complete dataset of toxin concentrations for all eight tissue types plus carcass. Toxin burdens for each tissue were calculated as the measured toxin concentration multiplied by the total weight of the collected tissue.

Fish size (biomass), growth rates (% increase in biomass), and accumulation of CTXs in the flesh (% of ingested toxin; i.e., 100 × muscle CTX burden/total CTX consumed) were compared with those of experimental fish from Clausing et al. [53]. Statistical comparisons were made using *t*-tests and were limited to those fish in the previous study that had also been exposed to the *G. polynesiensis*-enriched gel food for 16 weeks (*n* = 5). Rates of dosing, in proportion to fish biomass, were equivalent between studies, giving a larger total CTX dose in the present study in which fish were larger.

## Figures and Tables

**Table 1:** CTX-like activity in fish tissues as quantified by a radioligand-receptor binding assay (r-RBA) and the neuroblastoma cell-based assay (CBA-N2a). Distribution is given as tissue concentration (ng CTX3C equiv g^−1^ tissue equivalent (TE) in extract) and tissue burden (total ng CTX3C equiv), where tissue burden is calculated as a function of tissue concentration and total tissue weight (g). Data are reported as means ± SD and ranges, where available. For all values, *n* = 3–5 samples of the extract were analyzed. Given tissue and total body weights are the means for the selected fish analyzed, which differed between assays. The CTX concentrations and corresponding tissue burdens in the carcass by CBA-N2a (marked by *) are estimated from the relationship of other tissues with measures obtained by r-RBA. CBA-N2a totals include estimated carcass CTX loads (marked by **). Details of the calculation are found in the Supplementary Materials (Table S3). The smallest tissues were insufficient for quantification by r-RBA (marked by ***).

		r-RBA Assay				CBA-N2a Assay		
	
Tissue		Tissue Weight (g)	Concentration (ng CTX3C equiv g^−1^ TE)	Tissue Burden (ng CTX3C equiv)		Tissue Weight (g)	Concentration (ng CTX3C equiv g^−1^ TE)	Tissue Burden (ng CTX3C equiv)
spleen	mean ± SD	0.065 ± 0.018	98.06 ± 12.17	6.21 ± 0.85		0.049 ± 0.007	129.0 ± 34.9	6.47 ± 2.17
	min	0.053	84.05	5.62		0.04	106.02	4.41
	max	0.086	105.97	7.19		0.06	169.19	8.73
liver	mean ± SD	1.25 ± 0.29	18.09 ± 1.71	22.55 ± 5.43		1.39 ± 0.11	27.41 ± 11.30	38.25 ± 16.36
	min	0.89	15.84	15.98		1.29	14.36	19.87
	max	1.58	20.08	30.70		1.51	34.00	51.23
GI tract	mean ± SD	3.59 ± 0.53	9.87 ± 0.16	35.48 ± 5.77		3.22 ± 0.18	10.85 ± 7.22	34.78 ± 22.28
	min	3.12	9.76	30.52		3.11	4.35	13.57
	max	4.16	10.05	41.81		3.42	18.63	58.00
muscle	mean ± SD	27.64 ± 11.79	4.04 ± 0.79	114.0 ± 56.5		21.90 ± 1.60	9.13 ± 4.12	203.8 ± 101.4
	min	18.15	3.40	61.79		20.47	4.46	91.34
	max	46.26	5.34	177.01		23.62	12.21	288.38
gills	mean ± SD	1.18 ± 0.48	6.43 ± 0.93	7.52 ± 2.73		0.89 ± 0.13	3.29 ± 1.53	2.99 ± 1.50
	min	0.80	5.76	4.81		0.76	1.82	1.39
	max	2.02	7.97	11.70		1.03	4.88	4.35
eyes	mean ± SD		***			1.15 ± 0.065	1.47 ± 0.47	1.69 ± 0.55
	min					1.09	1.04	1.14
	max					1.22	1.97	2.24
brain	mean ± SD		***			0.260 ± 0.019	1.22 ± 0.87	0.330 ± 0.256
	min					0.25	0.41	0.10
	max					0.28	2.14	0.61
gall bladder	mean ± SD		***			0.090 ± 0.024	0.650 ± 0.12	0.061 ± 0.027
	min					0.07	0.56	0.04
	max					0.12	0.79	0.09
carcass	mean ± SD	28.97 ± 9.54	3.59 ± 0.55	102.4 ± 12.8		24.49 ± 3.12	3.98 ± 1.89 *	97.44 ± 12.44 *
	min	21.43	3.25	92.33		21.43		85.24 *
	max	49.35	4.22	116.9		27.68		110.1 *
viscera combined	mean ± SD	4.95 ± 0.73	12.9 ± 0.76	64.1 ± 12.5		4.75 ± 0.12	16.7 ± 7.77	79.6 ± 37.3
	min	4.49	12.0	54.1		4.65	8.56	39.8
	max	5.79	13.5	78.1		4.88	24.0	114.0
whole body	mean ± SD	59.36 ± 10.71	4.46 ± 0.38	283.84 ± 64.47		55.43 ± 1.44	7.00 ± 2.33 **	385.76 ± 120.84 **
	min	47.91	4.09	239.75		54.33	4.32 **	246.75 **
	max	73.77	4.85	357.83		57.06	8.57 **	465.72 **

GI tract = gastro-intestinal tract; viscera combined = liver + gastro-intestinal tract + spleen + gall bladder.

**Table 2:** Results of Tukey’s HSD post hoc testing comparing CTX-like activity among tissue types for (A) r-RBA and (B) CBA-N2a. For brevity, only significant comparisons are included. A full table including non-significant tests is included in the Supplementary Materials (Table S2).

		(A) r-RBA Assay					(B) CBA-N2a Assay			

Tissue 1	Tissue 2	Estimate	Lower CI	Upper CI	Adjusted *p*-Value		Estimate	Lower CI	Upper CI	Adjusted *p*-Value

brain	spleen						127.8	90.1	165.4	<0.001
eyes	spleen						−0.8	−38.5	36.8	<0.001
gallbladder	spleen						9.4	−28.3	47.0	<0.001
GI	spleen	88.2	78.5	97.8	<0.001		1.8	−35.8	39.5	<0.001
gills	spleen	91.6	83	100	<0.001		25.9	−11.7	63.6	<0.001
liver	spleen	80	71.3	88.6	<0.001		7.7	−30.0	45.3	<0.001
muscle	spleen	94	85.4	103	<0.001		127.5	89.9	165.2	<0.001
carcass	spleen	95.1	86.9	103	<0.001					
gills	liver	11.7	4.18	19.1	<0.001					
muscle	liver	14	6.57	21.5	<0.001					
carcass	liver	15.1	8.2	22	<0.001					

GI = gastro-intestinal tract.

**Table 3:** Relative CTX distribution among tissues, i.e., percent of total body burden, as determined by r-RBA and CBA-N2a (*n* = 3). The smallest tissues were unable to be analyzed by r-RBA due to insufficient quantity of extract. Carcasses were only quantified with r-RBA; carcass values by CBA-N2a are estimated based on the assumption of similar relative assimilation among tissues between the two methods (see Supplementary Materials Table S3 for details of calculation).

		M	L	G	GI	Sp	Gb	E	B	C
r-RBA	mean	37.52	8.75	2.49	12.64	1.93				37.30
	sd	10.25	0.54	0.20	1.38	0.51				9.38
CBA-N2a	mean	50.33	9.65	0.85	8.53	1.80	0.02	0.48	0.11	28.24
	sd	12.54	1.75	0.51	3.98	0.78	0.01	0.22	0.12	14.29
overall means	mean	43.93	9.20	1.67	10.59	1.85	0.02	0.48	0.11	32.77
	sd	12.41	1.26	0.96	3.49	0.61	0.01	0.22	0.12	11.90

M = muscle, L = liver, G = gills, GI = gastro-intestinal tract, Sp = spleen, Gb = gall bladder, B = brain, E = eyes, C = carcass.

**Table 4:** Toxicity accumulation in terms of percentage ingested CTXs retained in fish tissues as estimated from r-RBA and CBA-N2a data. Values were calculated based on an estimated mean total ingested toxin of 1280.4 ng fish^−1^. The smallest tissues were unable to be analyzed by r-RBA due to insufficient quantity of extract. Carcasses were only analyzed by r-RBA; carcass values by CBA-N2a are estimated based on the assumption of similar relative assimilation among tissues between the two methods (see Supplementary Materials Table S3 for details of calculation).

		M	L	G	GI	Sp	Gb	E	B	C	By Fish
r-RBA	mean	8.62	1.94	0.55	2.77	0.45	--	--	--	8.00	22.17
	sd	4.29	0.43	0.11	0.45	0.01				1.00	5.04
CBA-N2a	mean	15.91	2.99	0.23	2.72	0.51	0.005	0.132	0.026	7.61	30.13
	sd	7.92	1.28	0.12	1.74	0.17	0.002	0.043	0.020	0.97	9.44
overall means	mean	12.3	2.46	0.39	2.74	0.48	0.005	0.132	0.026	7.80	26.15
	sd	6.96	1.03	0.20	1.14	0.12	0.002	0.043	0.020	0.91	8.05

M = muscle, L = liver, G = gills, GI = gastro-intestinal tract, Sp = spleen, Gb = gall bladder, B = brain, E = eyes, C = carcass.

## Data Availability

The data presented in this study are available on request from the corresponding author.

## Supplementary Materials

The following supporting information can be downloaded at https://www.mdpi.com/article/10.3390/md22010014/s1, Figure S1: Experimental model for exposure to toxic microalgal cells: herbivorous fish graze on a gelatin-based food containing *Gambierdiscus* cells, solidified on a nylon mesh and attached to the aquarium wall with a plastic rod; Figure S2: Comparison of fish growth rate constants over time; Figure S3: Linear regression of fish growth over time; Figure S4: Total tissue burden as a function of body weight at time of sampling; Figure S5: Total CTX body burden as a function of body weight; Table S1: Growth of individual fish from each treatment; Table S2: Results of Tukey’s HSD post hoc testing comparing toxin concentrations among tissue types for r-RBA and CBA-N2a; Table S3: Calculation of estimated toxin concentration in remaining parts by N2A; Table S4: Asymptotic regression of fish CTX body burden by body mass; Table S5: Selected *m*/*z* transitions and instrument parameters for LC-MS/MS analysis.
