# Supplementary material for: Tissue Distribution and Metabolization of Ciguatoxins in an Herbivorous Fish following Experimental Dietary Exposure to Gambierdiscus polynesiensis
Source: Mar Drugs. 2023 Dec 25;22(1):14. doi: 10.3390/md22010014 (PMC10817614; doi:10.3390/md22010014)
Supplement: Supplementary file 1 [file marinedrugs-22-00014-s001.zip › marinedrugs-2773883-supplementary.pdf]

Supplementary materials:

Tissue distribution and metabolization of ciguatoxins in an herbivorous fish following experimental dietary exposure to *Gambierdiscus polynesiensis*

Rachel J. Clausing [1,2*], Hela Ben Gharbia [3], Khalil Sdiri [3], Manoëlla Sibat [4], Ma. Llorina Rañada-Mestizo [5], Laura Lavenu [3], Philipp Hess [4], Mireille Chinain [6] and Marie-Yasmine Dechraoui Bottein [7]

[1] Università degli Studi di Genova, Dipartimento di Scienze della Terra, dell'Ambiente e della Vita, 16132, Genova, Italia
[2] Department of Ecology and Evolutionary Biology, University of California, California, Los Angeles, USA
[3] International Atomic Energy Agency, IAEA Marine Environment Laboratories, 4 Quai Antoine 1er, 98000, Monaco, Monaco
[4] Ifremer, ODE/PHYTOX/METALG, Rue de l'île d'Yeu, F-44300 Nantes, France
[5] IAEA Collaborating Center on Harmful Algal Bloom (HAB) Studies, Chemistry Research Section, Philippine Nuclear Research Institute – Department of Science and Technology (DOST-PNRI), Diliman, Quezon City, 1101, Philippines
[6] Institut Louis Malardé- UMR 241 EIO, Laboratoire des Micro-algues Toxiques, BP 30, 98713, Papeete-Tahiti, French Polynesia
[7] Université Côte d'Azur, CNRS, ECOSEAS, UMR7035, Parc Valrose, CEDEX 2, 06103 Nice, France
* Correspondence: rachel.clausing@gmail.com;

Table S1. Growth of individual fish from each treatment.

Fish	Treatment	Initial weight	Final weight	Weight gain	Percent gain
1	**control**	44.2	62.5	18.4	41.6
2	**control**	30.9	52.8	22.0	71.2
3	**experimental**	36.2	54.9	18.7	51.6
4	**experimental**	23.4	47.9	24.5	105.0
5	**control**	45.2	61.8	16.6	36.8
6	**control**	28.9	59.7	30.8	106.7
7	**experimental**	36.2	58.7	22.5	62.2
8	**experimental**	32.3	57.1	24.8	76.8
9	**control**	47.1	53.7	6.6	14.1
10	**control**	25.7	48.9	23.2	90.3
11	**experimental**	38.0	73.8	35.8	94.4
12	**experimental**	31.0	54.3	23.3	75.0

Table S2. Results of Tukeys HSD post-hoc testing comparing toxin concentrations among tissue types for r-RBA and CBA-N2A.

		RBA assay				N2a-CBA assay			
**Tissue 1**	**Tissue 2**	estimate	lower CI	upper CI	adjusted *p*-value	estimate	lower CI	upper CI	adjusted *p*-value
remnants	**spleen**	95.1	86.9	103	**2.33E-14**	127.8	90.1	165.4	**6.50E-08**
muscle	**spleen**	94	85.4	103	**2.33E-14**	119.9	82.2	157.5	**1.61E-07**
liver	**spleen**	80	71.3	88.6	**2.33E-14**	101.6	63.9	139.2	**1.60E-06**
gills	**spleen**	91.6	83	100	**2.33E-14**	125.7	88.1	163.4	**8.20E-08**
GI	**spleen**	88.2	78.5	97.8	**2.33E-14**	118.2	80.5	155.8	**1.98E-07**
gallbladder	**spleen**					128.4	90.7	166.0	**6.09E-08**
eyes	**spleen**					127.5	89.9	165.2	**6.68E-08**
brain	**spleen**					127.8	90.1	165.4	**6.50E-08**
muscle	remnants	-1.07	-7.99	5.85	0.996				
liver	**remnants**	-15.1	-22	-8.2	**<0.001**				
gills	remnants	-3.47	-10.4	3.46	0.631				
GI	remnants	-6.9	-15.1	1.26	0.130				
liver	**muscle**	-14	-21.5	-6.57	**<0.001**	-18.3	-55.9	19.4	0.70
gills	muscle	-2.4	-9.87	5.08	0.914	5.8	-31.8	43.5	1.00
GI	muscle	-5.83	-14.5	2.8	0.322	-1.7	-39.4	35.9	1.00
gallbladder	muscle					8.5	-29.2	46.1	0.99
eyes	muscle					7.7	-30.0	45.3	1.00
brain	muscle					7.9	-29.7	45.6	1.00
gills	**liver**	11.7	4.18	19.1	**0.0009**	24.1	-13.5	61.8	0.39
GI	liver	8.22	-0.415	16.9	0.068	16.6	-21.1	54.2	0.79
gallbladder	liver					26.8	-10.9	64.4	0.28
eyes	liver					25.9	-11.7	63.6	0.31
brain	liver					26.2	-11.5	63.8	0.30
GI	gills	-3.43	-12.1	5.2	0.813	-7.6	-45.2	30.1	1.00
gallbladder	gills					2.6	-35.0	40.3	1.00
eyes	gills					1.8	-35.8	39.5	1.00
brain	gills					2.1	-35.6	39.7	1.00
gallbladder	GI					10.2	-27.5	47.9	0.98
eyes	GI					9.4	-28.3	47.0	0.99
brain	GI					9.6	-28.0	47.3	0.98
eyes	gallbladder					-0.8	-38.5	36.8	1.00
brain	gallbladder					-0.6	-38.2	37.1	1.00
brain	eyes					0.2	-37.4	37.9	1.00

Table S3. Calculation of estimated toxin concentration in remaining parts by N2A. A) Tissue:remaining parts ratio of toxin concentration as calculated by RBA for each tissue type. B) Estimated tissue:remaining parts ratio for N2A. C) Calculation of remaining parts by N2A, assuming a constant ratio between each tissue type and the measure of remaining parts between the two methods of analysis. D) A final estimate of the toxin concentration of remaining parts by N2A (mean ± SD) is reached by averaging values obtained for each tissue type (C).

**Tissue**	**ratio (A) = (B)**	**toxin ‰ r-RBA**	**toxin ‰ CBA-N2a**	**(C) est. carcass By CBA-N2a**
spleen	33.05	98.06	129.01	3.90
muscle	1.36	2.97	9.13	6.71
liver	6.10	4.04	27.41	4.50
gills	2.17	18.09	3.29	1.52
GI	3.33	6.43	10.85	3.26
			**mean ± SD**	**3.98 ± 1.89 (D)**

*

$$(A)\ \frac{TissueX_{RBA}}{Remaining\ parts_{RBA}} = \frac{TissueX_{N2A}}{Remaining\ parts_{N2A}}\ (B)$$

$$(C)\ Remaining\ parts_{N2A} = TissueX_{N2A} \times \frac{Remaining\ parts_{RBA}}{TissueX_{RBA}} \tag{1}$$

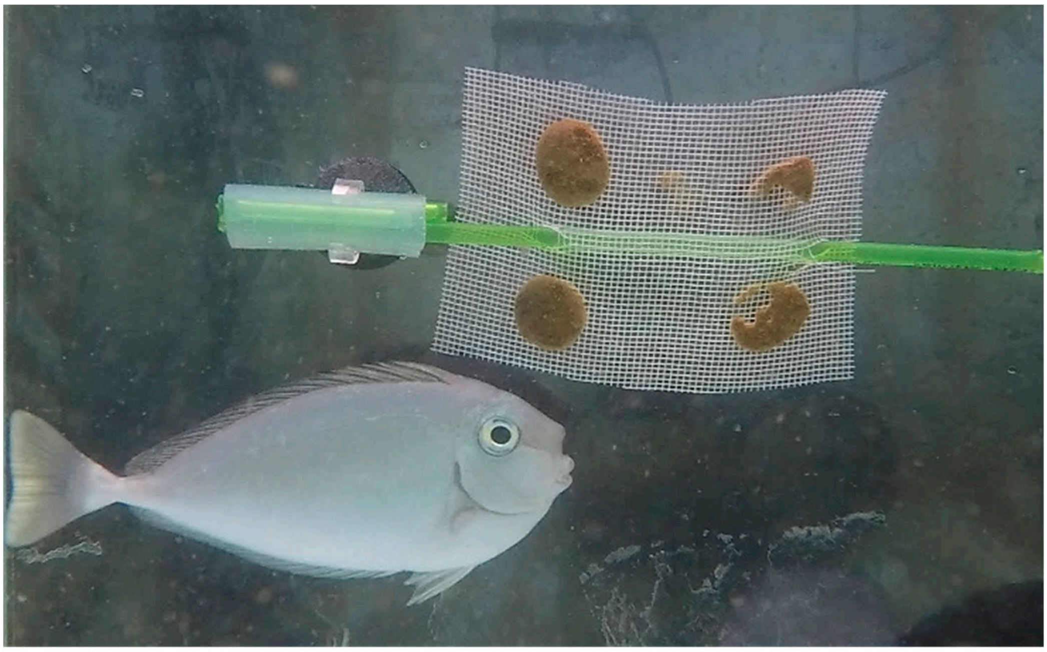

Figure S1. Experimental model for exposure to toxic microalgal cells: herbivorous fish graze on a gelatin-based food containing *Gambierdiscus* cells, solidified on a nylon mesh and attached to the aquarium wall with a plastic rod.

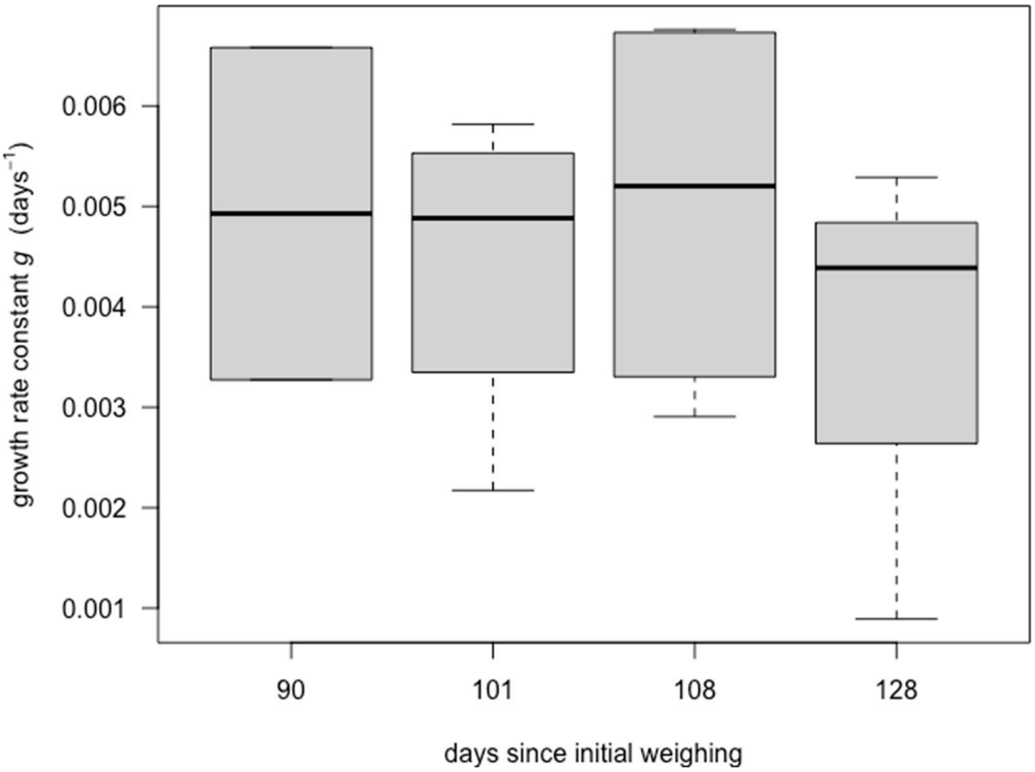

Figure S2. Comparison of growth rate constants (g) over time (following ln $W_t = W_{t=i} + gt$) where the x-axis is the date at which the growth rate was calculated for replicate individual fish (>3 weighing events for each fish). Each bar represents different replicate fish, with no overlap. The lack of significant differences over time validates the use of growth curves constructed with a subset of fish at points throughout the experiment to adjust the quantity of gel food offered with the goal of maintaining a constant toxin consumption rate.

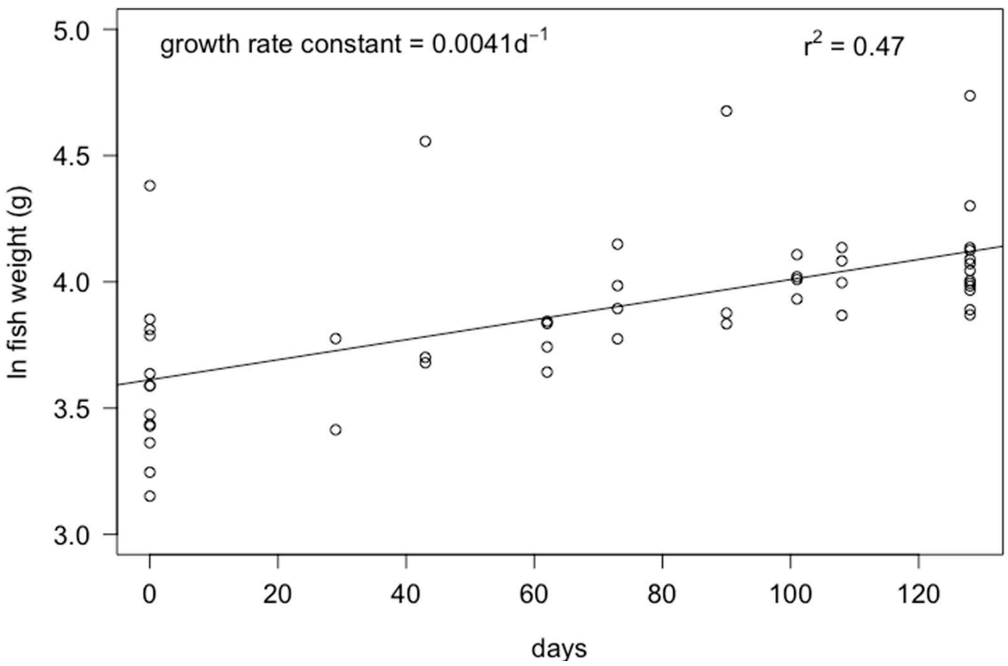

Figure S3. Linear regression of overall fish growth over time, from weight prior to tank distribution until sampling. The growth rate constant, g = 0.0041 d^{-1} is estimated by the slope of the curve fitted to ln W, the measured fish weights. Linear regression $p<0.001$, $R^2 = 0.47$.

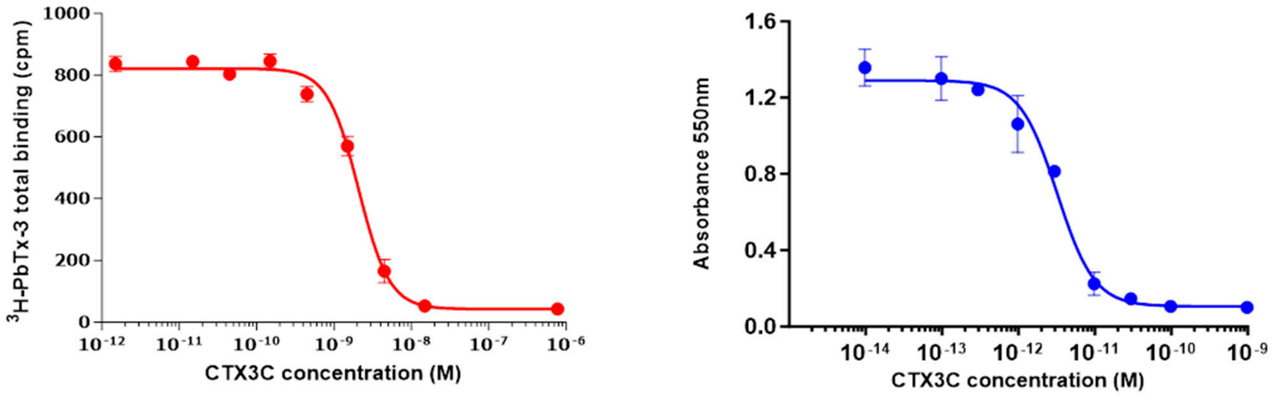

Figure S4. Sigmoidal standard curves of **(a)** PbTx3, obtained by radioligand-receptor binding assay (r-RBA) and (b) CTX3C, obtained by neuroblastoma cell-based assay (CBA-N2a).

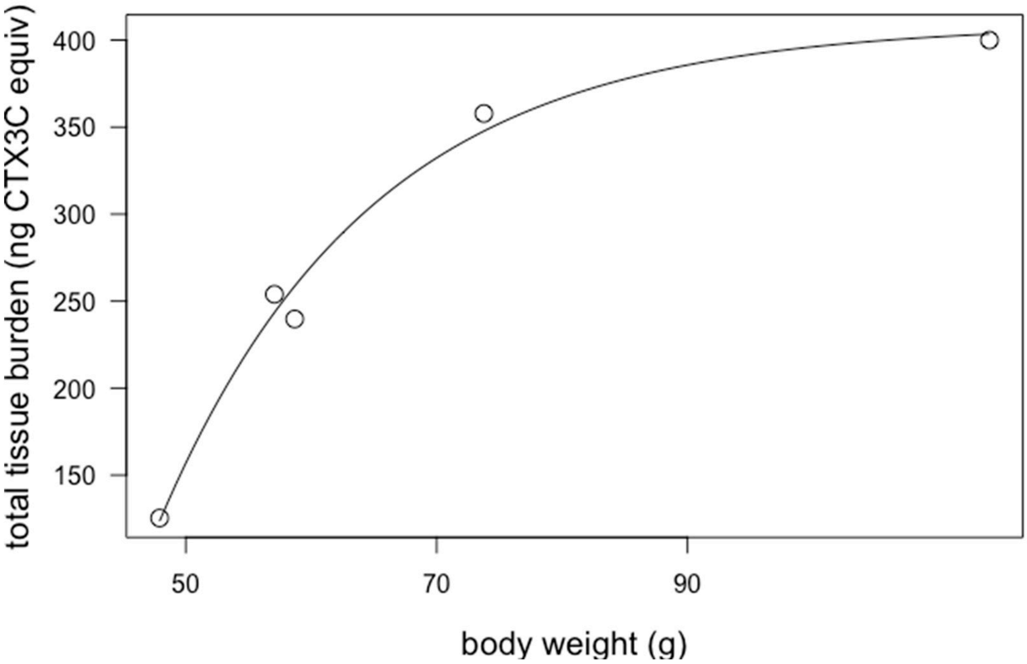

Figure S5. Total CTX body burden as a function of body weight at time of sampling. Toxin analysis by r-RBA. Data fit with an asymptotic regression.

Table S4. Model summary of asymptotic regression of total fish CTX body burden as a function of body mass (**Figure S4**).

parameter	estimate	SE	t-value	*p*-value
init:(Intercept)	-4530	2490	-1.82	0.210
m:(Intercept)	0.06	0.01	5.56	0.031
plateau:(Intercept)	409	18.7	21.9	0.002

Table S5: The selected *m*/*z* transitions and the LC-MS/MS instrument parameters used for the scheduled MRM method

Compound	Detection window (min)	Precursor ion (Q1) *m/z*	Product ion (Q3) *m/z*	DP (eV)	CE (eV)	CXP (eV)
CTX1B	3.1 ± 1	1128.6 $[M+NH_4]^+$	1093.6	105	20	12
			1075.6	105	30	12
			95.1	105	90	20
M-*seco*-CTX3C	4.7 ± 1	1041.6 $[M+H]^+$	1023.6	105	30	12
			1005.6	105	20	12
			125.1	105	50	18
2-OHCTX3C and 3-OHCTX3C	5.4 ± 1	1058.6 $[M+NH_4]^+$	1023.6	105	30	12
			1005.6	105	20	12
			125.1	105	50	18
2,3-diOHCTX3C	6.0 ± 1	1074.6 $[M+NH_4]^+$	1039.6	105	30	12
		1057.6 $[M+H]^+$	1039.6	105	20	12
			125.1	105	50	18
51-OHCTX3C	6.3 ± 1	1056.6 $[M+NH_4]^+$	1021.6	105	30	12
		1039.6 $[M+H]^+$	1021.6	105	20	12
			1003.6	105	20	12
M-*seco*-CTX4A/4B	6.5 ± 1	1096.6 $[M+NH_4]^+$	1043.7	105	30	12
		1079.6 $[M+H]^+$	1043.7	105	20	12
			125.1	105	50	18
52-*epi*-54-deoxyCTX1B and 54-deoxyCTX1B	6.8 ± 1	1112.6 $[M+NH_4]^+$	1077.6	105	20	12
			1059.6	105	30	12
			95.1	105	90	20
CTX3C isomers (1), (2) and (3)	7.6 ± 1	1040.6 $[M+NH_4]^+$	1005.6	105	30	12
		1023.6 $[M+H]^+$	1005.6	105	20	12
			125.1	105	20	12
CTX3C, 49-*epi*CTX3C and isomer (4)	10.5 ± 1	1040.6 $[M+NH_4]^+$	1005.6	105	30	12
		1023.6 $[M+H]^+$	1005.6	105	20	12
			125.1	105	50	18
CTX4A and CTX4B	12.2 ± 1	1078.6 $[M+NH_4]^+$	1043.6	105	30	12
		1061.6 $[M+H]^+$	1043.6	105	20	12
			125.1	105	50	18
